# Cell death sensitization of leukemia cells by opioid receptor activation

**DOI:** 10.18632/oncotarget.952

**Published:** 2013-04-16

**Authors:** Claudia Friesen, Mareike Roscher, Inis Hormann, Iduna Fichtner, Andreas Alt, Ralf A. Hilger, Klaus-Michael Debatin, Erich Miltner

**Affiliations:** ^1^ Center for Biomedical Research, University of Ulm, Ulm, Germany; ^2^ Institute of Legal Medicine, University of Ulm, Ulm, Germany; ^3^ Max Delbrueck Center for Molecular Medicine, Berlin, Germany; ^4^ Department of Internal Medicine, University of Essen, West German Cancer Center, Essen, Germany; ^5^ University Children's Hospital, University of Ulm, Ulm, Germany

**Keywords:** opioids, methadone, doxorubicin, cAMP, apoptosis, acute lymphoblastic leukemia

## Abstract

Cyclic AMP (cAMP) regulates a number of cellular processes and modulates cell death induction. cAMP levels are altered upon stimulation of specific *G-protein-coupled receptors* inhibiting or activating adenylyl cyclases. Opioid receptor stimulation can activate inhibitory G_i_-proteins which in turn block adenylyl cyclase activity reducing cAMP. Opioids such as D,L-methadone induce cell death in leukemia cells. However, the mechanism how opioids trigger apoptosis and activate caspases in leukemia cells is not understood. In this study, we demonstrate that downregulation of cAMP induced by opioid receptor activation using the opioid D,L-methadone kills and sensitizes leukemia cells for doxorubicin treatment. Enhancing cAMP levels by blocking opioid-receptor signaling strongly reduced D,L-methadone-induced apoptosis, caspase activation and doxorubicin-sensitivity. Induction of cell death in leukemia cells by activation of opioid receptors using the opioid D,L-methadone depends on critical levels of opioid receptor expression on the cell surface. Doxorubicin increased opioid receptor expression in leukemia cells. In addition, the opioid D,L-methadone increased doxorubicin uptake and decreased doxorubicin efflux in leukemia cells, suggesting that the opioid D,L-methadone as well as doxorubicin mutually increase their cytotoxic potential. Furthermore, we found that opioid receptor activation using D,L-methadone alone or in addition to doxorubicin inhibits tumor growth significantly *in vivo*. These results demonstrate that opioid receptor activation via triggering the downregulation of cAMP induces apoptosis, activates caspases and sensitizes leukemia cells for doxorubicin treatment. Hence, opioid receptor activation seems to be a promising strategy to improve anticancer therapies.

## INTRODUCTION

Acute lymphoblastic leukemia (ALL) is the most frequent malignant disease in children but affects adolescents as well [1]. The intensification of treatment regimens and advances in supportive care, improved the survival rates of childhood ALL to 80%, whereas in adults the overall survival rate remains at approximately 40% [2, 3]. Unfortunately, patients suffering from relapse have a poor outcome [4] which is mainly determined by the response to chemotherapy, involving a number of deregulated pathways like differentiation, survival and apoptosis [3, 5-7].

The main goal in chemotherapies is the concerted destruction of cells via apoptosis [8, 9]. Apoptosis can be mediated via the external death receptor/ligand pathway or the intrinsic pathway involving caspases [8-10]. Pro-apoptotic proteins like Bax and anti-apoptotic proteins (Bcl-2, Bcl-x_L_, XIAP) frequently involved in malignancies and treatment resistances regulate apoptosis induction [7, 11-14].

Previous studies showed that the second messenger cyclic AMP (cAMP) inhibits doxorubicin as well as DNA-damage-induced apoptosis [15, 16]. cAMP regulates a number of cellular processes. The production of cAMP is either increased or decreased upon stimulation of *G-protein-coupled receptors* which activate or inhibit adenylyl cyclases. cAMP is responsible for a multitude of actions like ion channel regulation and kinase activation [17-19]. Furthermore, cAMP can either stimulate or inhibit programmed cell death [20].

Methadone is a full-opioid agonist used as substitution for heroin or other opiates but also as long-lasting analgesic in cancer pain [21]. Opioid receptor activation initiates a cascade of events resulting in a diversity of biological effects like analgesis, sedation but also effects on cell survival and proliferation can be observed [22-25]. Opioid receptor stimulation can activate inhibitory Gi-proteins which in turn block adenylyl cyclase activity reducing cAMP [17].

The opioid D,L-methadone induces apoptosis in human T-lymphoblastic and myeloid leukemia cell lines and overcomes chemoresistance in leukemia cells without affecting healthy lymphocytes [25]. Singh et al found an effective synergism in cell death induction using D,L-methadone in addition to an anti-Bcl-2-agent [23]. Furthermore, D,L-methadone strongly inhibits proliferation of leukemia and human lung cancer cell lines [22, 25-27].

In this study, we found that opioid receptor activation induces cell death sensitization of leukemia cells *ex vivo* and *in vivo*. Our work provides evidence that the downregulation of cAMP induced by opioid receptor triggering induces apoptosis, activates caspases and sensitizes leukemia cells for doxorubicin treatment. In addition, we demonstrate that the opioid D,L-methadone-induced cell death depends on critical levels of opioid receptor expression which can be increased by doxorubicin. Additionally, D,L-methadone increases doxorubicin influx and hampers its efflux in leukemia cells.

## RESULTS

### D,L-Methadone induces cell death in xenograft-derived ALL cells depending on opioid receptor expression

**Figure 1:**
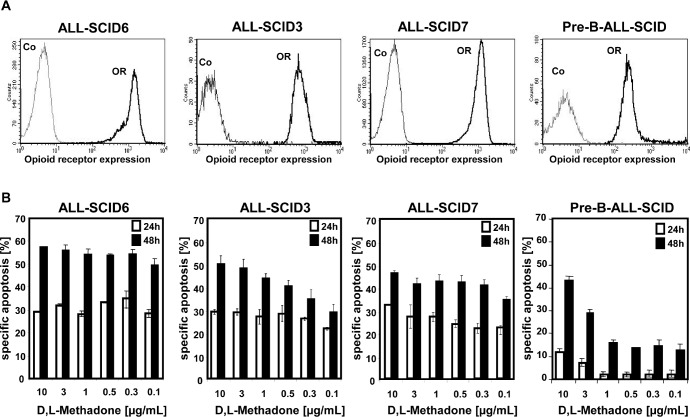
D,L-Methadone kills ALL cells *ex vivo* depending on critical levels of opioid receptor expression (a) Human ALL-SCID6, ALL-SCID3, ALL-SCID7, and pre-B-ALL-SCID leukemia cells derived from xenografted mice display different levels of opioid-receptors on their cell surface. The cells were stained with naloxone-fluoresceine measuring opioid-receptor expression (OR, thick black curve) and analyzed by flow cytometry. Controls (Co, unstained cells) are exhibited as thin black curves. (b) ALL-SCID6, ALL-SCID3, ALL-SCID7, and pre-B-ALL-SCID leukemia cells were treated with different concentrations of D,L-methadone (as indicated). After 24h (white columns) and 48h (black columns), the fractions of apoptotic cells were measured by FSC/SSC-analysis. The percentage of specific apoptosis was calculated as follows: 100 × [experimental dead cells (%) - spontaneous dead cells in medium (%)] / [100% - spontaneous dead cells in medium(%)]. Columns, mean of triplicates; bars, SD<10%.

The opioid D,L-methadone induces cell death in different leukemia cell lines [25]. To test the role of opioid receptor triggering in cell death induction and the clinical relevance of D,L-methadone in treatment of leukemia, we analyzed the anti-cancer effect of D,L-methadone in different xenograft-derived ALL cells. The xenografts were originally established from patients with T-cell (ALL-SCID6, ALL-SCID3), B-cell (ALL-SCID7) [28] and B-cell precursor (BCP, pre-B-ALL-SCID) acute leukemia. First, we measured opioid-receptor expression on xenograft-derived ALL cells. ALL-SCID6, ALL-SCID3, and ALL-SCID7 leukemia cells displayed high amounts of opioid-receptors whereas the pre-B-ALL-SCID leukemia cells expressed only moderate levels of opioid-receptors (Figure 1A). To analyze if cell death induction using D,L-methadone depends on the levels of opioid receptor expression, we treated the xenograft-derived ALL cells with different concentrations of D,L-methadone (Figure 1B). We used therapeutic plasma concentrations of D,L-methadone (≤ 3μg/mL) and included 10μg/mL D,L-methadone, because levels of D,L-methadone in lymphatic tissue and marrow might be higher [23]. Therapeutic plasma concentrations of D,L-methadone (≤ 3μg/mL) induced a strong cell death in xenograft-derived ALL cells (Figure 1B) expressing high amounts of opioid-receptors (Figure 1A). In contrast, the xenograft-derived-BCP-ALL cells (pre-B-ALL-SCID) expressing moderate opioid-receptor levels (Figure 1A) could only be slightly killed with therapeutic concentrations of D,L-methadone (Figure 1B). Therefore, apoptosis induction by D,L-methadone seems to depend on the level of opioid-receptor expression.

### D,L-methadone sensitizes ALL-cells for doxorubicin-induced cell death and caspase activation

**Figure 2:**
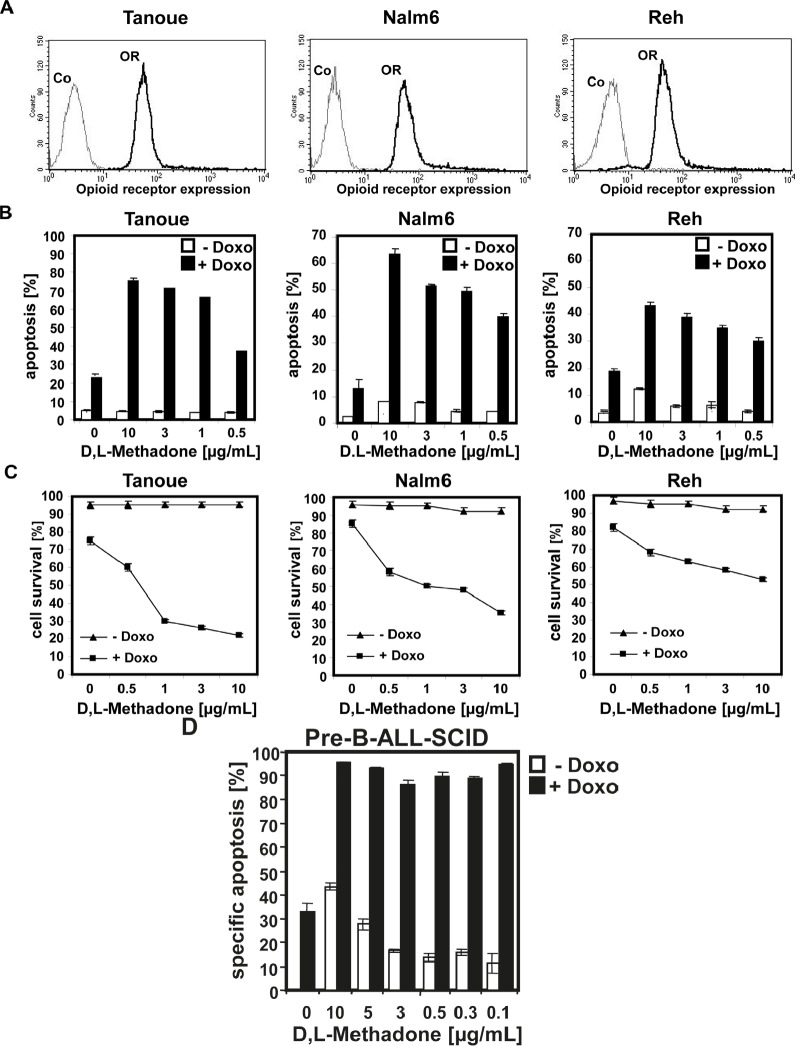
Combination treatment with D,L-methadone and doxorubicin induces apoptosis in ALL cells expressing moderate amounts of opioid receptors (a) Different BCP-ALL cell lines (Tanoue, Nalm6 and Reh) express a moderate number of opioid-receptors on their cell surface. Tanoue, Nalm6 and Reh were stained with naloxone-fluoresceine measuring opioid-receptor expression (OR, thick black curve) and analyzed by flow cytometry. Controls (Co, unstained cells) are exhibited as thin black curves. (b) BCP-ALL cell lines (Tanoue, Nalm6 and Reh) were treated with different concentrations of D,L-methadone alone (- Doxo, white columns), with doxorubicin alone or with D,L-methadone in addition to doxorubicin (+ Doxo, black columns). For the cell line Tanoue, we used doxorubicin in a concentration of 0.06μg/mL, for Nalm6 and Reh in a concentration of 0.01μg/mL. 120h after stimulation, the percentages of apoptotic cells were measured by FSC/SSC-analysis. (C) BCP-ALL cell lines (Tanoue, Nalm6 and Reh) were treated with different concentrations of D,L-methadone alone (- Doxo, triangle), with doxorubicin alone or with D,L-methadone in addition to doxorubicin (+ Doxo, square). For the cell line Tanoue, we used doxorubicin in a concentration of 0.06μg/mL, for Nalm6 and Reh in a concentration of 0.01μg/mL. 120h after stimulation, the percentages of surviving cells were measured by FSC/SSC-analysis (D). D,L-Methadone strongly enhances doxorubicin sensitivity of xenograft-derived-BCP-ALL-cells *ex vivo*. Xenograft-derived-BCP-ALL cells (pre-B-ALL-SCID) were treated with different concentrations of D,L-methadone (as indicated) alone (- Doxo, white columns), with 0.01μg/mL doxorubicin alone or with D,L-methadone in addition to doxorubicin (+ Doxo, black columns). 48h after stimulation, the percentages of apoptotic cells were measured by FSC/SSC-analysis. The percentage of specific apoptosis was calculated as described in Figure 1B. Columns, mean of triplicates; bars, SD<10%.

In analogous studies, we tested the cytotoxic potential of D,L-methadone on BCP-ALL cell lines (Tanoue, Reh, Nalm6) expressing opioid-receptors in a moderate level on their cell surface (Figure 2A). These BCP-ALL cell lines could only be killed slightly by D,L-methadone (Figure 2B) as observed for xenograft-derived-BCP-ALL cells (pre-B-ALL-SCID) (Figure 1B). As different substances can act synergistically, we treated Tanoue, Reh, Nalm6, and xenograft-derived-BCP-ALL cells (pre-B-ALL-SCID) with different concentrations of D,L-methadone and doxorubicin alone or in combination with each other (Figure 2 B, 2C and 2D). We observed that the combination treatment strongly killed the BCP-ALL cell lines (Figure 2B) and strongly reduced survival of BCP-ALL cell lines markedly (Figure 2C). The combination treatment also strongly killed xenograft-derived-BCP-ALL cells (pre-B-ALL-SCID) (Figure 2D).

**Figure 3:**
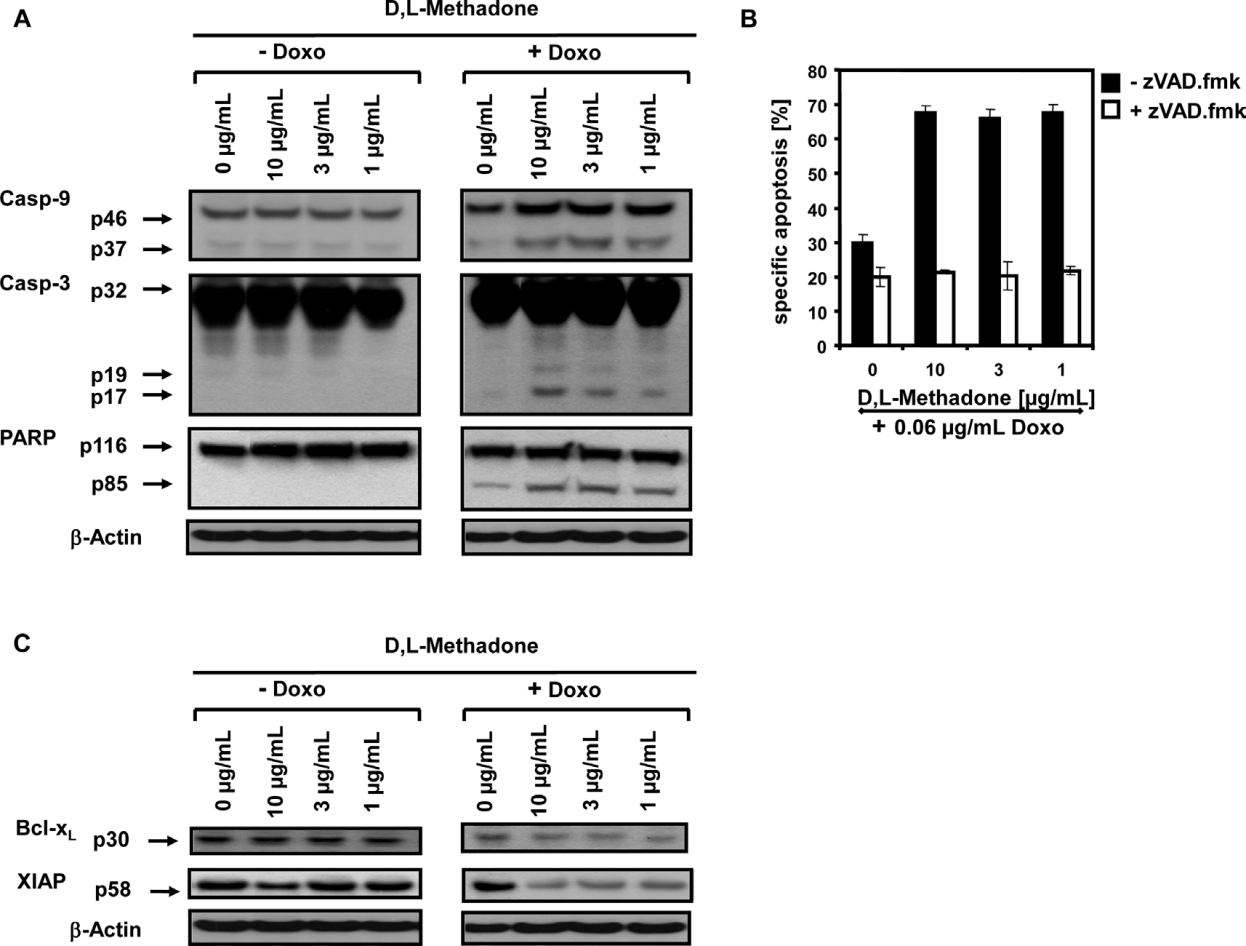
D,L-Methadone in combination with doxorubicin restores deficient activation of apoptotic pathways in BCP-ALL cells expressing moderate amounts of opioid receptors *in vitro* (a) D,L-Methadone and doxorubicin co-treatment provokes caspase activation. The BCP-ALL cell line Tanoue was treated with different concentrations of D,L-methadone (as indicated) alone (− Doxo), with 0.06μg/mL doxorubicin (+ Doxo) alone or with D,L-methadone in addition to doxorubicin (+ Doxo). After 120h, Western blot analyses for caspase-9, caspase-3 and PARP were performed. The active fragment of caspase-9 was detected at ~37 kDa, the active fragment of caspase-3 at ~19 kDa and ~17 kDa and PARP cleavage at ~85 kDa. Equal protein loading was controlled by anti-β-actin antibody. (b) D,L-Methadone and doxorubicin-induced apoptosis depends on caspase activation. Pre-incubation of the cell line Tanoue with 50μM of the caspase inhibitor zVAD.fmk for 1h (white columns, + zVAD.fmk,) or without pre-treatment (black columns, − zVAD.fmk,) was followed by addition of different concentrations of D,L-methadone (as indicated) in combination with 0.06μg/mL doxorubicin. Apoptosis induction was detected 120h after stimulation by FSC/SSC-analysis. The percentage of specific apoptosis was calculated as described in Figure 1B. Columns, mean of triplicates; bars, SD<10%. (c) Downregulation of XIAP and Bcl-x_L_ by D,L-methadone and doxorubicin co-treatment. The cell line Tanoue was treated with different concentrations of D,L-methadone (as indicated) alone (− Doxo), with 0.06μg/mL doxorubicin (+ Doxo) alone or with D,L-methadone in addition to doxorubicin (+ Doxo). After 120h, Western blot analyses for XIAP and Bcl-x_L_ were performed. XIAP was detected at ~58 kDa and Bcl-x_L_ at ~30 kDa. Equal protein loading was controlled by anti-β-actin antibody.

To analyze the molecular pathways of cell killing in more detail and to find out how the combination treatment with D,L-methadone and doxorubicin induced apoptosis, we analyzed which apoptotic effector molecules are activated in BCP-ALL cells upon this combination treatment compared to cells treated with D,L-methadone or doxorubicin alone. 120h after treating the BCP-ALL cell line Tanoue with D,L-methadone in addition to doxorubicin, we observed the activation of the caspase cascade in BCP-ALL cells. We found a strong activation of caspase-3 and caspase-9 and cleavage of the prototype substrate of caspase-3, PARP (Figure 3A).

The role of the caspase cascade in apoptosis induction was further investigated with the broad-spectrum inhibitor of caspases zVAD.fmk. zVAD.fmk strongly decreased cell death after combination treatment with D,L-methadone and doxorubicin in BCP-ALL cells (Figure 3B) underlining the dependence on caspase activation.

The apoptotic machinery is tightly controlled by anti-apoptotic factors like XIAP and Bcl-x_L_ [11, 12] which we found to be strongly downregulated in BCP-ALL cells treated with D,L-methadone in addition to doxorubicin (Figure 3C). These results indicate that the combination of D,L-methadone and doxorubicin sensitizes BCP-ALL cells for apoptosis via the activation of caspases and downregulation of XIAP and Bcl-x_L_.

### D,L-methadone enhances doxorubicin-uptake and inhibits doxorubicin-efflux whereas doxorubicin induces opioid-receptor expression

**Figure 4:**
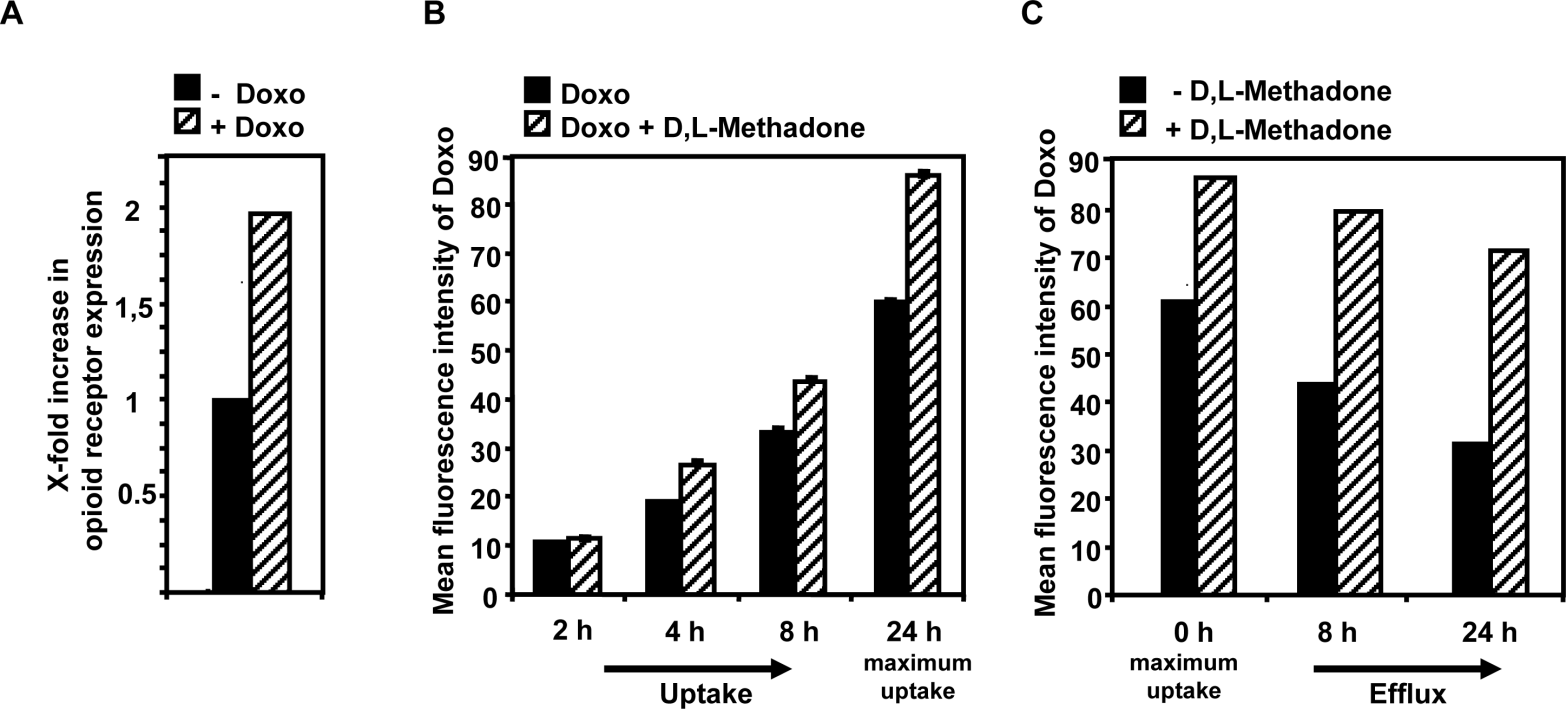
Doxorubicin enhances opioid receptor expression whereas D,L-methadone enhances doxorubicin uptake and inhibits its efflux (a) Doxorubicin enhances opioid receptor expression on the cells` surface. The BCP-ALL cell line Tanoue was treated for 96h with 0.06μg/mL doxorubicin. After staining of doxorubicin-treated (+ Doxo) and untreated cells (- Doxo) with naloxone-fluoresceine relative fluorescence intensities were determined flowcytometrically. X-fold increase in opioid receptor expression is shown after subtracting the cells autofluorescence (- Doxo) and doxorubicin fluorescence (+ Doxo). (b) D,L-methadone enhances doxorubicin uptake. The BCP-ALL cell line Tanoue was either pre-treated with 0.3μg/mL doxorubicin (black columns, Doxo) alone or with a combination of doxorubicin and 3μg/mL D,L-methadone (hatched columns, Doxo + D,L-Methadone) for 2h, 4h, 8h and 24h. Cell uptake was analyzed via doxorubicin fluorescence in cells using flow cytometry after 2h, 4h, 8h and 24h (maximum uptake). (C) D,L-Methadone inhibits doxorubicin efflux. 24h after maximal uptake of doxorubicin (0h, maximum uptake) cells were washed with medium to remove doxorubicin. After washing doxorubicin-treated BCP-ALL cells, BCP-ALL cells were either left untreated (black columns, - D,L-Methadone) or treated with 3μg/mL D,L-methadone (hatched columns, + D,L-Methadone) and incubated for different points in time (8h, 24h). Doxorubicin efflux was analyzed via doxorubicin fluorescence in cells using flow cytometry after 8h and 24h. Values are mean fluorescence intensities +/−SE.

The efficiency of cell death induction and activation of effector molecules in apoptosis pathways after treating leukemia cells with D,L-methadone seems to depend on the amount of opioid-receptors displayed on the cell`s surface. Combination treatment with D,L-methadone and doxorubicin kills leukemia cells with moderate opioid receptor expression, which could only be killed slightly by D,L-methadone or doxorubicin alone. Chemotherapeutics enhance the expression of receptors like CD95 in leukemia cells [29]. To analyze whether doxorubicin might influence the opioid-receptor expression, we treated the BCP-ALL cell line Tanoue with doxorubicin for 96h. Afterwards, the relative amount of opioid-receptors compared to untreated cells was measured by flow cytometry. We found that doxorubicin strongly increased opioid-receptor expression (Figure 4A) suggesting that D,L-methadone can bind in higher amounts to cells co-treated with doxorubicin.

Opioids as well as doxorubicin are substrates of the in multi-drug resistances-involved efflux pump P-glycoprotein (P-gp). Furthermore, D,L-methadone is known to inhibit P-gp [30-33]. To analyze whether D,L-methadone might influence the uptake and/or efflux of doxorubicin in leukemia cells, the BCP-ALL cell line Tanoue was incubated for different intervals with doxorubicin alone or with a combination of doxorubicin and D,L-methadone. After 4h, 8h, 24h (maximum uptake), we observed an enhanced doxorubicin concentration in the cells co-incubated with doxorubicin and D,L-methadone (Figure 4B). 24h after maximum uptake of doxorubicin, doxorubicin was removed from the supernatant and fresh medium was added without doxorubicin and D,L-methadone was applied. After 8h and 24h, D,L-methadone reduced the doxorubicin efflux strongly (Figure 4C) indicating that D,L-methadone increases doxorubicin uptake and inhibits doxorubicin efflux out of leukemia cells. This suggests that D,L-methadone as well as doxorubicin mutually increase their cytotoxic potential.

### Apoptosis induction by D,L-methadone and doxorubicin depends on opioid receptor activation inducing cAMP downregulation

**Figure 5:**
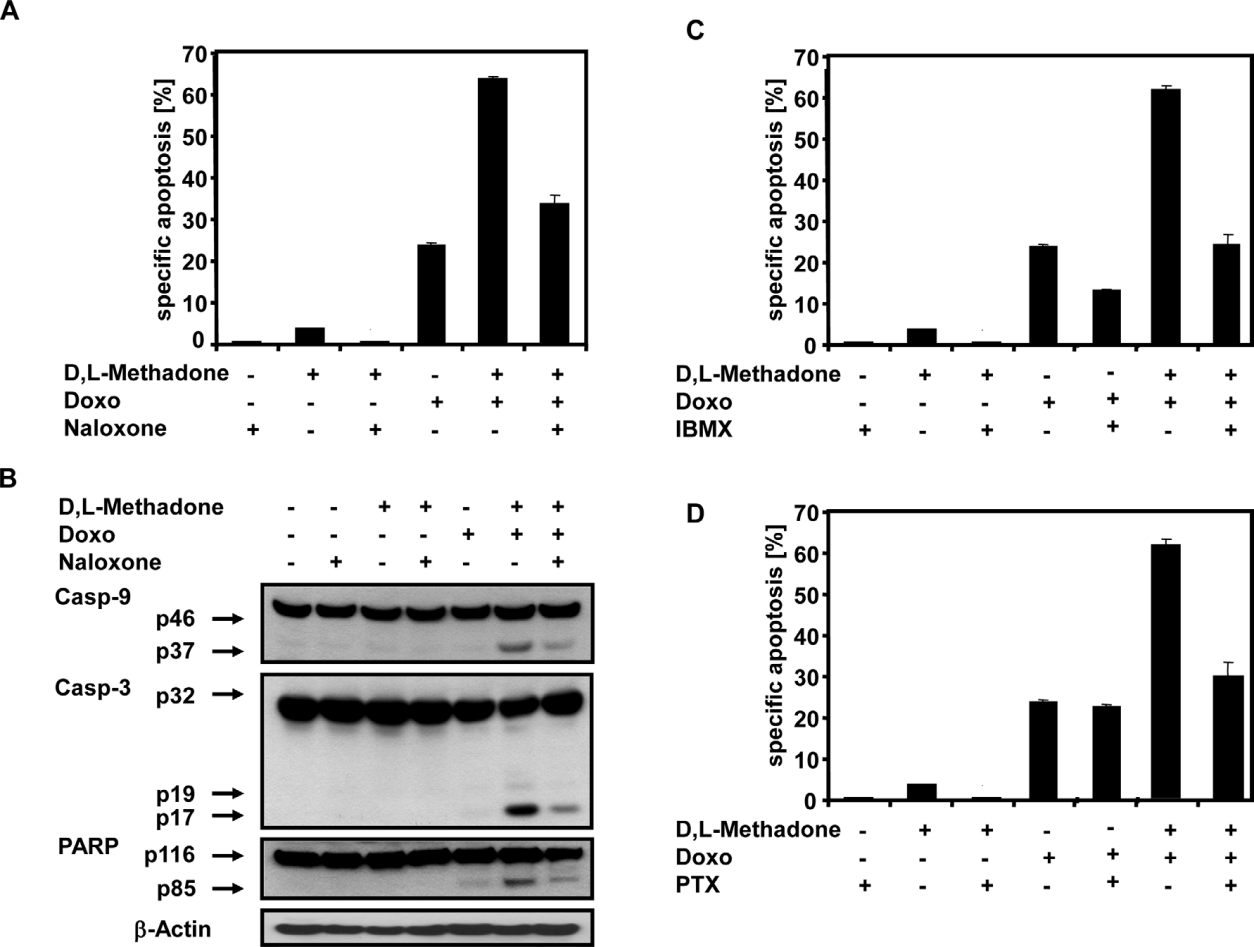
Combination treatment with D,L-Methadone and doxorubicin induced apoptosis depends on opioid-receptor triggering via downregulation of cAMP (a) Inhibition of opioid-receptor triggering inhibits apoptosis induction mediated by combination treatment with D,L-methadone and doxorubicin. The BCP-ALL cell line Tanoue was incubated with 60μg/mL naloxone (Naloxone), 3μg/mL D,L-methadone (D,L-Methadone) and 0.06μg/mL doxorubicin (Doxo) alone or in different combinations as indicated. After 96h, apoptotic cells were measured by FSC/SSC-analysis. (b) Inhibition of opioid-receptor triggering inhibits caspase activation mediated by combination treatment with D,L-methadone and doxorubicin. Tanoue cells were incubated with 60μg/mL naloxone (Naloxone), 3μg/mL D,L-methadone (D,L-Methadone) and 0.06μg/mL doxorubicin (Doxo) alone or in different combinations as indicated. Western blot analyses for caspase-9, caspase-3 and PARP were performed after 96h of incubation. The active fragment of caspase-9 was detected at ~37 kDa, of caspase-3 at ~19 kDa and ~17 kDa and PARP cleavage at ~85 kDa. Equal protein loading was controlled by anti-β-actin antibody. (c) Increasing cAMP levels via repression of phosphodiesterase activity inhibits apoptosis. Tanoue cells were incubated for 96h with 200μM 3-isobutyl-1-methylxanthine (IBMX), 3μg/mL D,L-methadone (D,L-Methadone) and 0.06μg/mL doxorubicin (Doxo) alone or in different combinations as indicated. (d) Uncoupling G_i_-proteins from opioid receptors inhibits apoptosis by preventing inhibition of adenylyl cyclases. Tanoue cells were incubated with 200ng/mL pertussis toxin (PTX), 3μg/mL D,L-methadone (D,L-Methadone) and 0.06μg/mL doxorubicin (Doxo) alone or in different combinations as indicated. After 96h, the percentages of apoptotic cells were measured by FSC/SSC-analysis. The percentages of specific apoptosis was calculated as described in Figure 1B. Columns, mean of triplicates; bars, SD<10%.

To further analyze the role of opioid-receptor triggering in apoptosis induction and consequently activation of apoptotic pathways, the BCP-ALL cell line Tanoue was treated with D,L-methadone, doxorubicin or with the opioid-receptor antagonist naloxone alone or in different combinations with each other (Figure 5A and 5B). After 96h, we found that blocking opioid-receptors by naloxone strongly reduced the apoptotic rates of the combination treatment with D,L-methadone and doxorubicin (Figure 5A). Additionally, naloxone co-treatment drastically reduced the activation of caspase-9 and caspase-3 and cleavage of PARP (Figure 5B) indicating that opioid-receptor triggering is involved in apoptosis induction and in caspase activation.

Opioid receptor stimulation activates inhibitory G_i_-proteins which in turn block adenylyl cyclase activity reducing cAMP (Figure 7) [18]. Pertussis toxin (PTX) inactivates G_i_-proteins and blocks downregulation of cAMP (Figure 7) [34]. IBMX, however, increases cAMP levels as a result of phosphodiesterase inhibition (Figure 7). To analyze the critical role of cAMP in opioid receptor activation-induced apoptosis, the BCP-ALL cell line Tanoue was treated with D,L-methadone, doxorubicin, and IBMX or PTX either alone or in different combinations with each other (Figure 5C and 5D). After 96h we found that upregulation of cAMP by IBMX (Figure 5C) as well as blocking downregulation of cAMP by PTX (Figure 5D) strongly reduced the apoptotic rates of combination treatment with D,L-methadone and doxorubicin. In addition, the upregulation of cAMP by IBMX also decreased doxorubicin-induced apoptosis (Figure 5C). These results indicate that the activation of G^i^-protein-coupled opioid receptors is essential for the induction of apoptosis which might be regulated via the intracellular cAMP levels.

### Inhibition of tumor growth *in vivo*

**Figure 6:**
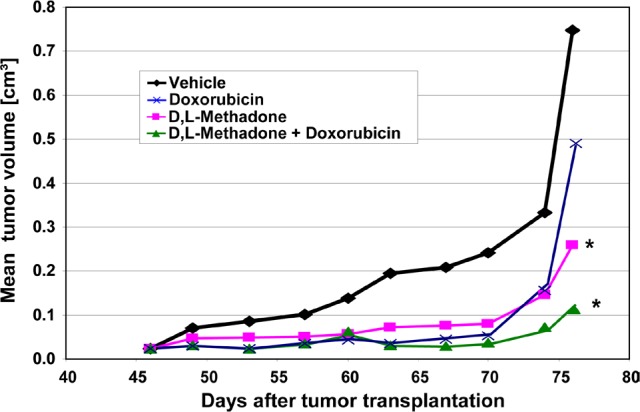
Opioid receptor activation by D,L-methadone inhibits growth of leukemia xenografts and increases doxorubicin sensitivity *in vivo* Fragments of an *in vivo* passage of a patient-derived T-ALL (ALL-SCID6, see also Figure 1) were transplanted into male NSG mice. Mice were treated with D,L-methadone alone (n=8, orally day 1-76, D,L-Methadone), with doxorubicin alone (n=8, i.v. day 46,53,60,76, Doxorubicin) or with a combination treatment with D,L-methadone and doxorubicin (n=8, D,L-Methadone + Doxorubicin). D,L-Methadone was used in weekly increasing doses from 20 up to 120mg/kg/day and doxorubicin in a dose of 3mg/kg. As control group xenografted mice were treated i.v. with 10% Tween 80 in saline (n=8, Vehicle). For 76 days after transplantation all mice were monitored for tumor growth, body weight and health condition. *significant to vehicle (p<0.05, Mann-Whitney U test).

*In vitro* results demonstrated that D,L-methadone induces apoptosis in several leukemia cell lines and increases the cytotoxicity of doxorubicin. To confirm the clinical relevance of the anti-cancer potential of D,L-methadone alone or in combination with doxorubicin and to verify the results obtained so far an ALL-xenograft study was undertaken (Figure 6).

For the *in vivo* study, a patient-derived ALL-xenograft model (ALL-SCID6) was used. Its phenotypic and genotypic identity with the original patient sample was proven [28]. The experiment started with subcutaneous inoculation of ALL-SCID6 fragments from an in vivo passage into male NOD/SCID/IL2rγ null (NSG) mice. After randomization, D,L-methadone was orally administered after ALL-inoculation with increasing doses. When tumors were palpable, doxorubicin treatment was initiated. D,L-Methadone and doxorubicin treatment led to a significant inhibition of tumor growth at comparable levels (Figure 6). Combination treatment with D,L-methadone and doxorubicin had a similar anti-tumor efficacy as D,L-methadone or doxorubicin alone until day 70 (Figure 6). At later time points, the tumor inhibition was longer lasting during the combined treatment of D,L-methadone and doxorubicin (Figure 6). The therapy was well-tolerated with body weight changes of -10% for the combination and -8% or -4% for the D,L-methadone or doxorubicin treatment, respectively. To analyze D,L-methadone serum concentrations in mice, 0.5h, 1h, 4h and 24h after the last D,L-methadone application, serum was taken and D,L-methadone quantified by mass spectrometry. The serum concentrations of methadone were found between 56ng/mL and 230ng/mL in the time course of 0.5h until 4h after D,L-methadone application. The serum concentrations of doxorubicin were found between 156ng/mL and 198ng/mL. These results demonstrate that D,L-methadone and the co-treatment using doxorubicin and D,L-methadone significantly inhibited tumor growth *in vivo*.

## DISCUSSION

ALL is a malignant disorder originating from single B- or T-lymphocyte progenitors [1]. Despite the significant progress made in the overall cure rate, the prognosis of relapsed ALL patients still remains poor [2] as cellular resistances to anti-cancer drugs occur [3, 6, 7, 35-37]. Hence, it is important to find alternate therapies overcoming resistances. Recent studies demonstrate that D,L-methadone kills leukemia cells in vitro and inhibits cell proliferation of lung cancer cells [22, 23, 25-27]. Additionally, D,L-methadone abrogates chemoresistance in leukemia cell lines without affecting normal lymphocytes [25]. Co-treatment with D,L-methadone and an anti-Bcl-2-agent leads to synergistic effects in leukemia cells [23]. However, the underlying mechanisms how D,L-methadone leads to apoptosis induction are not understood. Additionally, the tumor growth inhibitory potential of D,L-methadone has not been demonstrated *in vivo* nor have the synergistic effects in cell killing with conventionally administered chemotherapeutics been analyzed. In this study, we provide evidence that D,L-methadone induces apoptosis, activates caspases and increases doxorubicin-triggered cell death in leukemia cells after opioid-receptor activation inducing the downregulation of cAMP. In addition, we demonstrate first that D,L-methadone can strongly reduce tumor growth of ALL *in vivo*.

Methadone binds as agonist to mu-opioid receptors. We found that D,L-methadone kills xenograft-derived ALL cells expressing high levels of opioid receptors. In contrast, D,L-methadone induces cell death only slightly in xenograft-derived ALL cells and cell lines expressing moderate opioid receptor amounts indicating that D,L-methadone-induced apoptosis depends on critical levels of opioid receptor expression in leukemia cells.

Combination treatment may prove to be advantageous in malignancies that still partially respond to either treatment alone as different therapeutics are known to interact with each other amplifying weaker death signals [2, 5, 35, 38-43]. Combination treatment with D,L-methadone and doxorubicin enhances the anti-tumor efficacy of both agents synergistically in BCP-ALL cells expressing moderate levels of opioid-receptors and increases caspase activation playing a critical role in apoptosis induction in sensitive and resistant cancer cells [6]. Furthermore, the downregulation of the anti-apoptotic proteins XIAP and Bcl-xL involved in the occurrence of resistances in many malignancies like ALL or NHL [44-48] is markedly enhanced. These suggest that combination treatment of D,L-methadone and doxorubicin increases apoptosis induction, caspase activation and downregulation of XIAP and Bcl-x_L_ synergistically.

Resistance to conventional chemotherapeutic drugs is a limiting factor of therapies whereby multidrug resistances resulting from overexpression of drug transporters such as P-gp are well-characterized [33, 49]. While in healthy cells the P-gp expression belongs to the normal cellular defense system, in cancer cells the overexpression of P-gp correlates with decreased survival and poor outcome [50, 51]. D,L-Methadone could be shown to be a substrate of P-gp [33, 49] inhibiting its action [30-32]. We found that co-treatment of doxorubicin with D,L-methadone enhances doxorubicin uptake and furthermore inhibits doxorubicin-efflux out of leukemia cells, suggesting that D,L-methadone sensitizes leukemia cells for doxorubicin-induced apoptosis by increasing concentrations of doxorubicin within the cells.

Posovsky et al found that chemotherapeutic drugs like doxorubicin sensitize BCP-ALL cells expressing low amounts of CD95 receptors on their surface for CD95-mediated apoptosis and caspase activation by upregulating CD95 receptors [29]. The enhanced toxicity of the combination treatment of D,L-methadone and doxorubicin is associated with an increased expression of opioid-receptors after doxorubicin treatment. Therefore, D,L-methadone can bind in higher amounts to cells co-treated with doxorubicin. These results indicate that the enhanced toxicity in the combination treatment with D,L-methadone and doxorubicin is associated with the upregulation of opioid-receptor expression mediated by doxorubicin and furthermore with an increased uptake and decreased efflux of doxorubicin mediated by D,L-methadone.

**Figure 7:**
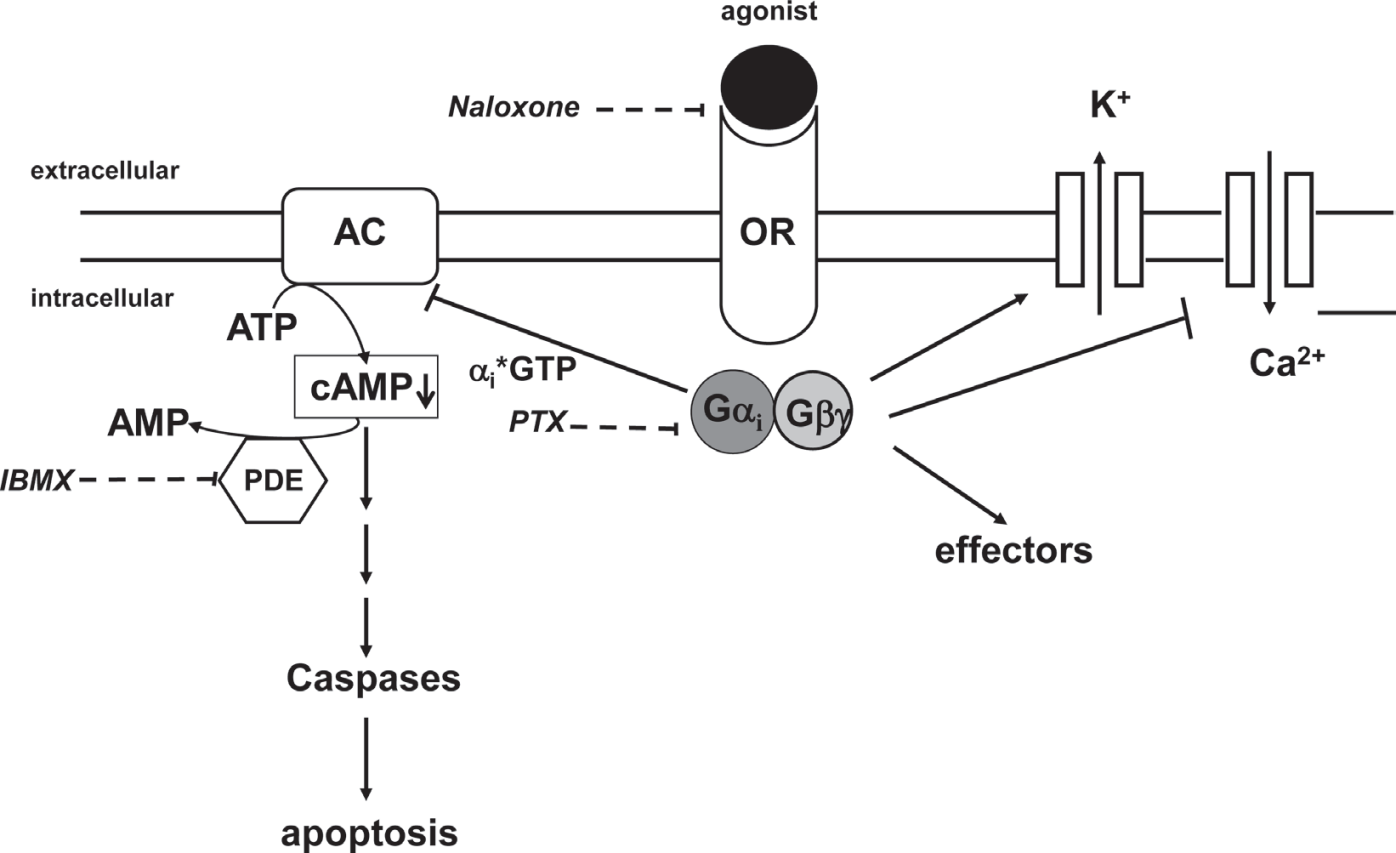
Opioid receptor signaling Stimulation of opioid receptors (OR) by agonists like D,L-methadone leads to an activation of the inhibitory G_i_-protein. The alpha_i_-subunit inactivates adenylyl cyclases (AC) resulting in a reduction of cAMP levels within the cell which in turn leads to apoptosis. Naloxone as opioid receptor antagonist inhibits competitively opioid receptors. PTX (pertussis toxin) inactivates G_i_-proteins and blocks downregulation of cAMP. IBMX (3-isobutyl-1-methylxanthine) inhibits phosphodiesterase activity (PDE) and increases cAMP levels.

Opioid receptors signal by catalyzing ligand-dependent nucleotide exchange on G_i_, thereby inhibiting adenylyl cyclase and modulating N-type calcium channels as well as G-protein–gated inwardly rectifying potassium (GIRK) channels leading to changes in cell signaling (Figure 7) [52]. The dependence of apoptosis induction on opioid-receptor triggering is underlined by their inhibition. Blocking opioid-receptor signaling with the opioid receptor antagonist naloxone inhibited combination treatment with D,L-methadone and doxorubicin-induced apoptosis and caspase activation in a high rate, suggesting that opioid-receptor triggering by D,L-methadone is involved in apoptosis induction and caspase activation (Figure 7). Maneckjee et al showed for human lung cancer cells that the opioid receptor antagonist naloxone in combination with methadone increased cAMP levels suggesting that inhibition of opioid receptor activation by methadone rises cAMP [26]. The second messenger cAMP is involved in a number of physiologic functions in response to various extracellular stimuli controlling cell proliferation, differentiation, and apoptosis whereby it can either inhibit or stimulate apoptosis dependent on the respective cell type [20, 53]. For various tumor cells like pancreatic or leukemia cells stimulated with different agents, it could be demonstrated that cAMP elevation is associated with impaired cell death [54-56]. Responsible for this protective action of cAMP against apoptosis is among others the synthesis of anti-apoptotic proteins, inactivation of pro-apoptotic proteins, and activation of PI3K-dependent Akt [57-59]. In the AML cell line HL-60, for instance, PKA inhibitors impair the cytoprotective effect from cAMP on the cells [60]. Activation of cAMP plays also a critical role in inhibiting DNA-damage- and doxorubicin-induced apoptosis via p53 dephosphorylation [15, 16] and furthermore by NF-κB activation [61]. Opioid receptor stimulation activates inhibitory Gi-proteins which in turn block adenylyl cyclase activity reducing cAMP [17]. We found that D,L-methadone induced apoptosis and activated caspases by triggering opioid receptors via downregulation of cAMP. Blocking the activation of opioid receptors by naloxone or inhibiting G_i_-proteins with PTX increased cAMP and strongly reduced apoptotic signaling triggered by the co-treatment with D,L-methadone and doxorubicin. In addition, upregulation of cAMP by inhibition of cAMP phosphodiesterases using IBMX reduced the cytoxicity of the combination treatment using D,L-methadone and doxorubicin in human BCP-ALL cells. Furthermore, opioid receptor triggering by D,L-methadone inhibits proliferation and leads to accumulation of leukemia cells in G1-phase which was previously observed [25]. These results suggest that opioid receptor activation by D,L-methadone triggers downregulation of cAMP mediating caspase activation and apoptosis induction in leukemia cells (Figure 7) and sensitizes leukemia cells for doxorubicin treatment.

*In vitro* results have shown that D,L-methadone can induce apoptosis in several leukemia cell lines [23, 25]. In the current study, we could verify the clinical relevance with patient-derived ALL cells *ex vivo* and we provide the first evidence that D,L-methadone as monotherapy or in combination with doxorubicin leads to a strong tumor growth inhibition in a patient-derived leukemia model *in vivo*. Both the anti-leukemic efficacy and the side effects of D,L-methadone alone or in combination with doxorubicin were comparable with those of doxorubicin alone. Anyhow, a longer lasting growth inhibition could be proven upon combination treatment. The serum concentrations of methadone in mice correlated with the concentrations showing *in vitro* cytotoxicity. This indicates that D,L-methadone may have a promising therapeutic potential in leukemia therapy and could improve therapeutic success of conventional therapies.

Taken together, our results demonstrate that D,L-methadone induces apoptosis, mediates caspase activation and sensitizes leukemia cells for doxorubicin treatment through opioid receptor activation triggering the downregulation of cAMP (Figure 7). D,L-methadone-induced cell death depends on critical levels of opioid receptor expression which can be increased by doxorubicin. Additionally, D,L-methadone increases doxorubicin influx and hampers its efflux in leukemia cells. Furthermore, we could demonstrate for the first time that D,L-methadone alone or in combination with doxorubicin leads to a significant tumor growth inhibition in a patient-derived leukemia model *in vivo*. These results have important implications for the development of novel strategies in cancer therapy especially when conventional therapies are less effective.

## METHODS

### Drugs and reagents

For the *in vitro* experiments, D,L-methadone hydrochloride (D,L-methadone) and doxorubicin were purchased from Sigma (Taufkirchen, Germany), naloxone from Fagron GmbH&Co. KG (Barsbüttel, Germany), and pertussis toxin (PTX) from Calbiochem (Nottingham, UK). Prior to each experiment these substances were freshly dissolved in sterile distilled water to ensure the constant quality of the preparations. 3-Isobutyl-1-methylxanthine (IBMX, Sigma) was freshly dissolved in 0.01N NaOH.

For *in vivo* application, we used D,L-methadone (Methaddict, Hexal, Germany) as 5mg tablets purchased from the local pharmacy. The tablets were pulverized and solubilized freshly before use in 10% Tween 80 in saline. Doxorubicin (Hexal) was purchased as injection solution (5mg/mL) and diluted freshly with saline to the appropriate concentrations.

### Cell lines

The human B-cell leukemia (BCP-ALL) cell lines Tanoue, Reh and Nalm6 were obtained from the DSMZ (Braunschweig, Germany) and cultured in RPMI 1640 (Invitrogen) containing 10% FCS (Lonza, Verviers, Belgium), 1mmol/L glutamine (Invitrogen), 1% penicillin/streptomycin (Invitrogen), 25mmol/L HEPES (Biochrom) at 37°C, 95% air/ 5% CO2. In experimental settings, the leukemia cells were seeded in a density of 100000 cells/mL.

### Serum concentrations of methadone

Determination of methadone in serum samples was carried out after liquid/liquid extraction using a mass spectrometer equipped with a gas chromatograph (GC/MS). As internal standard d9-methadone was added. The mass selective detector was operated in electron impact mode. Data were acquired in the selected-ion monitoring mode. The analytes were identified with the following masses m/z 294, 223, 72 (target ion) for methadone and m/z 303, 226, and 78 for d_9_-methadone with a limit of detection of 0.8ng/mL and a limit of quantification of 1.2ng/mL.

### Serum concentrations of doxorubicin

Determination of doxorubicin and its main metabolites in serum were performed as described previously [62, 63]. Using this validated method, the quantification of doxorubicin, doxorubicinol, and 7-deoxy-doxorubicinolon was possible with a LLQ of 0.2ng/mL.

### Patient-derived ALL xenografts

For *in vivo* use the ALL-SCID6 model was chosen. Fragments from in vivo passaged tumors were transplanted subcutaneously at day 0 to 32 male NOD/SCID/IL2rγ null (NSG) mice. After randomization oral treatment (by gavage) with D,L-methadone was initiated one day later and performed daily until the end of the experiment with increasing doses: 1^st^week 20mg/kg/d, 2^nd^week 30mg/kg/d, 3^rd^week 40mg/kg/d, 4^th^week 60mg/kg/d, 5^th^-10^th^week 2x60mg/kg/inj. The dose adaptation was necessary to avoid toxic deaths because of an overdosage of D,L-methadone. The maximum tolerated dose of D,L-methadone in the employed mouse strain is 2x60mg/kg/inj. At day 46, 53, 60 and 76 doxorubicin (3mg/kg) was administered i.v. Tumor size was measured twice weekly at two dimensions and tumor volumes were calculated according to the formula (length × width^2^)/2. Mean tumor volumes and standard deviations were calculated per group. Treated to control values (T/C) in percent were calculated by relating mean tumor volumes of each group at each measurement day to the controls. Individual body weight was determined twice per week as parameter for tolerability. Body weight changes [%] were calculated by relating the mean values of each group to the first measurement day.

Serum from D,L-methadone treated mice was taken 0.5, 1, 4 and 24 hours after last D,L-methadone treatment at day 76, respectively, and stored at -20°C until the determination of methadone concentration. Mice were sacrificed at day 77 for ethical reasons.

For the *in vitro* investigations, cell suspensions of human xenograft-derived ALL cells from patients with T-cell (ALL-SCID6, ALL-SCID3), B-cell (ALL-SCID7) and B-cell precursor (BCP, pre-B-ALL-SCID) acute leukemia were gained and cultivated *in vitro* and were phenotypically and genotypically characterized as described [28]. All animal experiments were approved by the local responsible authorities (LaGeSo Berlin) and performed according to the guidelines for animal welfare in oncological experiments [64].

### Flow cytometric assay for determination of cell surface opioid-receptors

Cells were washed in PBS supplemented with 1% FCS, centrifuged and resuspended in PBS/1% FCS containing naloxone-fluoresceine (0.05mM, Invitrogen) [65]. After 30min of incubation at RT, the cells were washed, centrifuged and resuspended in icecold PBS/1% FCS. Flow cytometry analysis was performed using FACSCalibur (BD, Heidelberg, Germany).

### Induction of apoptosis and determination of cell survival

ALL cells were treated with D,L-methadone (≤ 3μg/mL therapeutic plasma concentration) alone or in addition to doxorubicin in 175cm^2^ flasks or 96-well plates. Further experiments were performed simultaneously after addition of 60μg/mL naloxone, 200μM IBMX (3-isobutyl-1-methylxanthine) or 200ng/mL PTX (pertussis toxin). After different points in time, apoptosis rates were measured by flow cytometry [66, 67]. To determine apoptosis, cells were lysed with Nicoletti-buffer containing sodium citrate (0.1%), Triton X 100 (0.1%) and propidium iodide (50μg/mL) as described by Nicoletti [67]. Apoptotic cells were determined by hypodiploid DNA (subG1) or forward scatter/side scatter analysis [66]. Cell survival was determined by forward scatter/side scatter analysis using flow cytometry [66]. The percentage of specific apoptosis was calculated as follows: 100 × [experimental dead cells (%) - spontaneous dead cells in medium (%)] / [100% -spontaneous dead cells in medium (%)]. The spontaneous dead cells were in the range of 5 to 10% using cell lines. The spontaneous dead of untreated human xenograft-derived ALL cells was less than 35% at 24h and 48h.

### General caspase inhibition by zVAD.fmk

For inhibition of apoptosis, leukemia cells were treated with the pancaspase inhibitor zVAD.fmk (z-Val-Ala-D,L-Asp-fluoromethylketone; Bachem, Bubendorf, Germany) as described [68, 69]. 50μM zVAD.fmk was added to the cells 1h before stimulation with D,L-methadone and doxorubicin. The percentage of apoptotic cells was determined by FSC/SSC analysis via flow cytometry [66].

### Western blot analysis

Western blot analyses were performed as described [37, 68-70]. Whole cell lysates were immunodetected for PARP, caspase-3, caspase-9, XIAP, Bcl-x_L_ and ß-actin using rabbit-anti-PARP-polyclonal-antibody (1:5000, Roche), anti-XIAP-monoclonal-antibody (both 1:1000, BD Transduction Laboratories, Heidelberg, Germany), mouse-anti-caspase-3-monoclonal-antibody, rabbit-anti-caspase-9-polyclonal-antibody (both 1:1000, Cell Signaling, Boston, MA, USA) rabbit-anti-Bcl-x_L_-polyclonal-antibody (1:1000, Santa-Cruz, Heidelberg, Germany) and mouse-anti-ß-actin-monoclonal-antibody (1:5000, Sigma). As secondary antibodies peroxidase-conjugated-goat-anti-mouse IgG or peroxidase-conjugated-goat-anti-rabbit IgG (1:5000, Santa-Cruz) were used for the enhanced chemoluminescence system (ECL, Amersham-Pharmacia, Freiburg, Germany). Equal protein loading was controlled by ß-actin detection.

### Analysis of doxorubicin uptake and efflux

For analysis of doxorubicin uptake, the BCP-leukemia cell line Tanoue was seeded in a density of 100000 cells/mL in 175cm^2^ flasks and was either left untreated or incubated with 0.3μg/mL doxorubicin or a combination of 0.3μg/mL doxorubicin and 3μg/mL D,L-methadone. After different time point in time cells were washed and the relative doxorubicin uptake in cells was analyzed using flow cytometry.

For analysis of doxorubicin efflux, cells were washed to remove doxorubicin from medium after incubation for 24h (maximum uptake). Next, cells were incubated with fresh medium without doxorubicin or fresh medium containing 3μg/mL D,L-methadone without doxorubicin to measure doxorubicin efflux. After different time points, cells were harvested, washed and relative doxorubicin content in leukemia cells was analyzed using flow cytometry.
